# EEG-based classification of alzheimer’s disease and frontotemporal dementia using functional connectivity

**DOI:** 10.1038/s41598-026-35316-9

**Published:** 2026-01-09

**Authors:** Tjaša Mlinarič, Arne Van Den Kerchove, Zoe I. Barinaga, Marc M. Van Hulle

**Affiliations:** 1 Laboratory for Neuro- and Psychophysiology, Department of Neurosciences, KU Leuven, Leuven, Belgium; 2 Leuven Brain Institute, KU Leuven, Leuven, Belgium; 3 University of Lille, CNRS, Centrale Lille, Lille, France

**Keywords:** Electroencephalography, Functional connectivity, Alzheimer’s disease, Frontotemporal dementia, Classification, Manifold geometry, Computational biology and bioinformatics, Diseases, Neurology, Neuroscience

## Abstract

Alzheimer’s disease (AD) and frontotemporal dementia (FTD) are two major causes of dementia, with overlapping clinical and pathophysiological characteristics. We investigated whether resting-state EEG functional connectivity could distinguish AD, FTD, and healthy controls (HC) using a stacked ensemble learning approach. A publicly available dataset (openneuro.org/datasets/ds004504) was used to extract multiple connectivity metrics across frequency bands. Base classifiers were trained using the Fisher’s Geodesic Minimum Distance to Mean (FgMDM) approach with Euclidean or Riemannian distance metrics, and a meta-classifier was trained on their cross-validated predictions. Using leave-one-subject-out cross-validation, the stacked model achieved ROC-AUC values of 81.80% and 71.36% for distinguishing AD and FTD from HC, respectively. This was lower than the best single-base classifier performance, where several connectivity metrics achieved ROC-AUC values of 85% or higher for distinguishing AD and FTD. AD-FTD discrimination proved most challenging (ROC-AUC of 65.10%), suggesting overlapping network disruptions that limit separability. Classification performance was highest in the alpha band when distinguishing AD and FTD from HC, whereas the highest performance for distinguishing AD from FTD was observed in the delta band. Overall, these findings highlight the potential of EEG connectivity features for dementia classification and emphasize the importance of careful feature engineering and low-complexity models in the case of small-sample datasets.

## Introduction

Alzheimer’s disease (AD) and frontotemporal dementia (FTD) are two of the most prevalent forms of dementia^1^, posing significant challenges for patients, their caregivers, and the healthcare system. Both dementias exhibit characteristic patterns of progressive brain atrophy, but overlapping symptoms and high individual variability in disease presentation and progression make it difficult to clinically differentiate between the two^2,3^.

AD accounts for 60–80% of global dementia cases and typically presents with memory and communication problems, mood and behavioral changes, and poor judgment^1^. As the disease progresses, these conditions worsen, placing significant socio-economic burdens on family members and the healthcare system^4^. In contrast, FTD currently accounts for up to 10% of dementia cases in individuals younger than 65^1^ and is characterized by changes in personality and behavior and impairments in cognitive functioning, with pathological changes in the frontal and temporal lobes^5^.

Given the clinical and pathophysiological similarities between AD and FTD, validated biomarkers would be valuable in improving the accuracy of diagnosis, particularly in the early stages when treatments can be most effective^3^. In addition to clinical evaluations and imaging or fluid-based biomarkers, electroencephalography (EEG) has recently gained considerable attention due to its non-invasive nature, cost-effectiveness, and accessibility^3^. EEG measures brain electrical activity and has been utilized to characterize differences between AD and FTD. Patients with AD typically show an overall slowing of the signal over time, with decreased power in higher frequencies and increased power in lower frequencies^6,7^. Furthermore, they exhibit changes in functional connectivity (FC) parameters compared to age-matched controls without dementia and patients with FTD^8^. In contrast, patients with FTD show changes in the spectral power ratio between the frontal and temporal lobes^9^ and decreased power in higher frequencies^5,10^, although these findings are less consistent across studies, with some showing the opposite pattern^11^. These findings suggest that EEG connectivity analyses may provide complementary information to structural imaging or fluid-based biomarkers^12^.

To improve the diagnostic accuracy for patients with AD and FTD, several studies have utilized various EEG features in combination with machine learning algorithms to differentiate between these dementias and healthy controls (HC). Using a recently published dataset of 88 participants^13^, including patients with AD, FTD, and HC, different groups achieved classification accuracies ranging from 77.01% to 95.86% for distinguishing AD from HC^13–15^. However, distinguishing between AD and FTD is more challenging, given the overlapping characteristics between two dementias. For differentiating between AD and FTD, Wang et al.^16^ used a combination of spectral and aperiodic features, achieving an area under the receiver operating characteristic curve (ROC-AUC) of 73% with a Support Vector Machine (SVM). Si et al.^14^ and Rostamikia et al.^17^ used FC-based features and achieved an ROC-AUC of 91% (81% accuracy) with a Gaussian Naive Bayes classifier^14^ and 88% accuracy with an SVM approach^17^. However, both studies segmented the data into epochs and used k-fold cross-validation as a testing method. Table 1 summarizes the studies that used the mentioned dataset to distinguish between the two dementias. The AD/FTD/HC classification problem is a diagnosis problem, requiring a single prediction per subject and it is not clear how these studies aggregated epoch-based predictions into a reliable prediction on a subject-level. Furthermore, EEG signals are subject-specific, and including epochs from the same subject in both the training and test sets allows classifier to rely on subject-specific patterns instead of disease-related characteristics^18^. Proper separation of subject data across sets is therefore needed to avoid data leakage. These methodological inconsistencies highlight the importance of subject-level evaluation and controlled evaluation methods.

Studies using FC-based approaches to distinguish between AD, FTD, and HC have typically used Euclidean distance to quantify the dissimilarity between data points, represented by connectivity metrics extracted from EEG signals. However, connectivity matrices are typically high-dimensional and symmetric positive definite (SPD), and Euclidean distance does not account for their intrinsic manifold geometry, making selection of the appropriate distance metric crucial for accurately assessing their dissimilarity^19^. Barachant et al.^19^ showed the possibility of using a Riemannian geometry-based framework to more accurately quantify dissimilarity between SPD matrices. While their results were mainly focused on the classification of covariance matrices in a brain-computer interfacing (BCI) context, Corsi et al.^20,21^ showed that the same concepts can be applied to different connectivity metrics. Their FUCONE approach^22^ proposes to use an ensemble classifier over multiple SPD connectivity metrics, each separately classified on the Riemannian manifold.

Building on this framework, we design a stacked ensemble approach combining multiple connectivity features across different frequency bands. While the approach we propose shares core design principles with the FUCONE framework, it differs in several aspects. We introduce an initial filter-based feature selection step and apply an additional wrapper-based feature selection step. Next, we adapt the framework to between-subject decoding by aggregating epoch-level predictions into a subject-level probability. Finally, we explore the use of Euclidean and log-Euclidean distance metrics besides the affine-invariant Riemannian distance metric. With this approach we aim to accurately identify patients with AD and FTD from HC and to differentiate between AD and FTD, while also identifying features contributing most to classification performance.

**Table 1:** Overview of previous work on the same dataset.

Study	Preprocessing	Features	Evaluation	Problem	Classifier	Acc.	Sens.	Spec.	F1	AUC
Chen et al.^23^	Bandpass 0.5–45 Hz, artifact rejection, epochs	Spectral power, time domain	Test split	AD/HC FTD/HC AD/FTD	CNN+ transformer	85.78 80.36 79.03	83.22 76.34 77.47	81.76 79.77 80.12	- - -	85.88 81.77 81.05
Si et al.^14^	Bandpass 0.5–45 Hz, artifact rejection, epochs	FC, graph features	5-fold CV	AD/HC FTD/HC AD/FTD	GNB	86.2 84.7 81.1	90.0 87.0 81.0	- - -	86.0 84.4 78.9	90.7 90.7 90.1
Lal et al.^24^	Bandpass 0.5–45 Hz, artifact rejection, epochs	SVD entropy	15-fold CV SMOTE	AD/HC FTD/HC AD/FTD	kNN	91 93 91	- - -	- - -	- - -	- - -
Wang et al.^16^	Bandpass 0.5–45 Hz, artifact rejection, CAR	Spectral power, aperiodic component	Monte-Carlo CV, SMOTE	AD/FTD	SVM	-	-	-	-	73
Zandbagleh et al.^25^	Downsample, CAR, bandpass 1–45 Hz, artifact rejection, epochs	Multiscale dispersion entropy, PSD	LOSOCV	AD/HC FTD/HC AD/FTD	LR	-	77.78 56.52 75.00	79.31 79.31 69.57	- - -	85 67 62
Ma et al.^26^	Bandpass 0.5–45 Hz, artifact rejection, epochs	Mutual information, age, sex	LOSOCV	AD/HC FTD/HC AD/FTD	SVM	76.9 90.4 91.5	- - -	72.0 87.9 86.7	75.4 90.2 91.3	- - -
Rostamikia et al.^17^	Bandpass 0.5–45 Hz, artifact rejection	Time domain, frequency domain, FC, complex features	10-fold CV	AD + FTD/HC AD/FTD	SVM	93.5 87.8	90.0 85.1	93.0 90.0	- -	- -
Zheng et al.^27^	Bandpass 0.5–45 Hz, artifact rejection	Recurrence complexity, Hurst exponent, rate gradient	LOSOCV	AD/HC FTD/HC AD/FTD AD + FTD/HC	SVM	87.69 82.69 72.88 86.36	- - - -	75.86 89.66 39.13 72.41	- - - -	- - - -
Mouazen et al.^28^	Bandpass 0.5–45 Hz, artifact rejection, epochs, standardization	Spectral power, entropy features	k-fold CV	AD/FTD	Bi-LSTM	98	99	99	99	-
Current study	Bandpass 0.5–45 Hz, artifact rejection, CAR, epochs	Multiple FC measures	LOSOCV	AD/HC FTD/HC AD/FTD	Stacked ensemble	73.85 71.15 69.49	69.44 69.57 73.91	79.31 72.41 66.67	74.63 68.09 65.38	81.80 71.36 65.10
Current study	Bandpass 0.5–45 Hz, artifact rejection, CAR, epochs	α-CSD α-Cross-covariance δ-Cross-correlation	LOSOCV	AD/HC FTD/HC AD/FTD	FgMDM	84.62 82.69 64.41	77.78 73.91 52.21	93.10 89.66 72.22	84.85 79.07 53.33	88.70 86.36 70.65

Studies using the public dataset by Miltiadous et al.^13^ focusing on the AD/FTD problem are summarized.

AD = Alzheimer’s disease, AUC = Area under the curve, Bi-LSTM = Bidirectional long short-term memory, CAR = Common average reference, CNN = Convolutional neural network, CSD = Cross-spectral density, CV = Cross-validation, FC = Functional connectivity, FgMDM = Fisher’s geodesic minimum distance to mean, FTD = Frontotemporal dementia, GNB = Gaussian naïve Bayes, HC = Healthy controls, kNN = K-nearest neighbours, LOOCV = Leave-one-out cross-validation, LOSOCV = Leave-one-subject out cross-validation, LR = Logistic regression, PSD = Power spectral density, SMOTE = Synthetic minority oversampling technique, SVD = Singular value decomposition, SVM = Support vector machine.

## Methods

### Participants

The dataset of Miltiadous et al.^13^ included 88 participants, of which 36 were patients with AD, 23 patients with FTD, and 29 age-matched healthy controls. Their cognitive status was evaluated with the Mini-Mental State Examination (MMSE), which ranges from 0 to 30, with lower score indicating more severe cognitive impairment. Table 2 shows demographic information per group and statistical comparison between groups.

**Table 2:** Demographic information.

	AD (*n* = 36)	FTD (*n* = 23)	HC (*n* = 29)	Statistic
Age, years	66.4 ± 7.9 (49, 79)	63.6 ± 8.2 (44, 78)	67.9 ± 5.4 (57, 78)	*F*(2,85) = 2.22; *p* = 0.115
Sex, female, *n*(%)	24 (67)	9 (39)	11 (38)	***χ***^***2***^ **(2) = 6.78;** ***p*** **= 0.034**
MMSE	17.75 ± 4.5 (4, 23)	22.17 ± 2.6 (18, 27)	30 ± 0 (30, 30)	***F*(2**,**85) = 119.73;** ***p*** **< 0.001**

Values for age and MMSE are presented as mean ± SD (range). Statistical comparisons were performed using one-way ANOVA for age and MMSE, and a chi-square test for sex.

AD = Alzheimer’s disease, FTD = Frontotemporal dementia, HC = Healthy controls, MMSE = Mini Mental State Examination.

### EEG acquisition and signal processing

EEG was recorded using 19 electrodes, positioned according to the 10–20 international system, with two additional electrodes placed on the mastoids. The signal was recorded during resting state with eyes closed using a sampling rate of 500 Hz. Recording time varied in length across groups. On average, recordings lasted 13.5 min (min = 5.1, max = 21.3) for the AD group, 12 min for the FTD group (min = 7.9, max = 16.9), and 13.8 min (min = 12.5, max = 16.5) for the HC group. The study in which this data was collected was approved by the Scientific and Ethics Committee of AHEPA University Hospital, Aristotle University of Thessaloniki.

We made use of the available preprocessed version of the dataset, which was filtered with a Butterworth bandpass filter between 0.5 and 45 Hz. In the preprocessed version, to remove large artifacts, Artifact Subspace Reconstruction was used, followed by an independent component analysis to reject eye and jaw related artifacts. Additionally, data was re-referenced to a common average reference and cut into non-overlapping epochs of five seconds, with bad channels or trials interpolated or rejected with the Autoreject pipeline^29^. This resulted in 5445 epochs for the AD group, 3182 epochs for the FTD group, and 3182 epochs for the HC group.

Additionally, we applied 4th-order Butterworth filters to the preprocessed epochs to obtain five distinct sets corresponding to the frequency bands: delta (0.5–4 Hz), theta (4–8 Hz), alpha (8–14 Hz), beta (14–30 Hz), and gamma (30–45 Hz).

### Functional connectivity feature extraction

For each frequency band, multiple SPD FC matrices were calculated as described below. In total, this resulted in 5 × 12 = 60 matrix features, each with dimensionality 19 × 19 (number of channels × number of channels).

If the calculated matrices deviated slightly from the SPD constraint, due to a numerical error or an underdetermined estimation, they were projected onto the SPD manifold by finding the nearest SPD matrix using the method proposed by Higham^30^.

#### Time domain metrics

Let us model two band-pass filtered EEG channels as stochastic signals $X$ and $Y$.

Covariance is a straightforward time-domain FC metric and the most frequently used metric in Riemannian geometry analysis of neural signals. It measures the linear joint variability between two signals and is defined as

$$\mathrm{Cov}\left(X,Y\right)=\mathrm{E}\left[\left(X-\mathrm{E}\left[X\right]\right)\left(Y-\mathrm{E}\left[Y\right]\right)\right]$$

Pearson correlation is the normalized form of covariance, given by

$$\mathrm{Corr}\left(X,Y\right)=\frac{\mathrm{Cov}\left(X,Y\right)}{\sqrt{\mathrm{Var}\left(X\right)\hspace{0.17em}\mathrm{Var}\left(Y\right)}}$$

with $\:\mathrm{Var}\left(X\right)\hspace{0.17em}=\:\mathrm{E}\left[{\left(X-\mathrm{E}\left[X\right]\right)}^{2}\right]$ denoting the variance of signal $X$.

Cross-covariance determines the covariance when a time shift is introduced between signals $X$ and $Y$ and accounts for time-lagged effects. Instead of selecting one or more fixed lags, we chose the lag that maximizes the absolute cross-covariance:

$$\mathrm{XCov}\left(X,Y\right)={\left[X\times Y\right]}_{\mathrm{argmax}\left(\left|X\times Y\right|\right)}$$

Cross-correlation is the normalized form of the cross-covariance:

$$\mathrm{XCorr}\left(X,Y\right)=\frac{\mathrm{XCov}\left(X,Y\right)}{\sqrt{\mathrm{Var}\left(X\right)\hspace{0.17em}\mathrm{Var}\left(Y\right)}}$$

#### Frequency domain metrics

Let $\:{S}_{XY}=\mathcal{F}\{X\times\:Y\}$, where $\:\mathcal{F}$ denotes the discrete Fourier-transform. ${S}_{XY}$ is the cross-spectral density of $X$ and $Y$, limited to the frequency range of the frequency band of interest. Then the cross-spectral density magnitude is defined as

$$\mathrm{CSD}\left(X,Y\right)=\left|{S}_{XY}\right|$$

The cross-spectral density expresses how consistently two signals are phase-locked and share power at the frequencies of interest.

Coherence, a commonly used FC metric, is defined as the normalized squared cross-spectral magnitude:

$$\mathrm{Coh}\left(X,Y\right)=\frac{{\left|{S}_{XY}\right|}^{2}}{{S}_{XX}{S}_{YY}}$$

#### Information domain metrics

Mutual information measures the statistical dependence between two variables.

When modeling $X$ and $Y$ as stochastic variables, their entropy $\mathrm{H}\left(X\right)$ and conditional entropy $\mathrm{H}(X \ | \ Y)$ can be determined. Their mutual information is then defined as

$$\mathrm{MI}\left(X,Y\right)=\mathrm{H}\left(X\right)-\mathrm{H}(X \ | \ Y)$$

Entropy was estimated using empirical probability distributions obtained from histograms, with the number of bins per subject determined using the Freedman-Diaconis rule^31^ for data-driven estimation. Analogous to other metrics, a normalized form can be defined, the entropy correlation coefficient

$$\mathrm{ECC}\left(X,Y\right)=\frac{\mathrm{MI}\left(X,Y\right)}{\sqrt{\mathrm{H}\left(X\right)\hspace{0.17em}\mathrm{H}\left(Y\right)}}$$

*Analytical domain*.

Using the Hilbert transform, a real-valued signal $X$ can be transformed into the complex-valued analytical signal ${X}_{\mathrm{H}}$. The amplitude envelope of this analytical signal, given by its magnitude, describes the fluctuation in energy within the selected frequency band.

The amplitude envelope covariance between two signals is defined as

$$\mathrm{AECov}(X,Y) = \mathrm{Cov}\left( \left| X_{\mathrm{H}} \right|, \left| Y_{\mathrm{H}} \right| \right)$$

This metric can be normalized, yielding amplitude-envelope correlation, a commonly used measure of amplitude coupling:

$$\mathrm{AECorr}(X,Y) = \mathrm{Corr}\left( \left| X_{\mathrm{H}} \right|, \left| Y_{\mathrm{H}} \right| \right)$$

Finally, the angle of the analytical signal indicates the phase evolution of a signal. Using circular statistics, the phase locking value (PLV) quantifies the strength of phase-to-phase coupling:

$$\mathrm{P}\mathrm{L}\mathrm{V}\left(X,Y\right)=\left|\mathrm{E}\left[{e}^{i(\mathrm{a}\mathrm{r}\mathrm{g}{X}_{\mathrm{H}}-\mathrm{a}\mathrm{r}\mathrm{g}{Y}_{\mathrm{H}})}\right]\right|$$

The amplitude-weighted PLV incorporates the magnitudes of the analytic signals to reduce the influence of phase-locking when power is low:

$$\mathrm{w}\mathrm{P}\mathrm{L}\mathrm{V}\left(X,Y\right)\:=\:\frac{\left|\mathrm{E}\left[\left|{X}_{\mathrm{H}}\right|\left|{Y}_{\mathrm{H}}\right|{e}^{i(\mathrm{a}\mathrm{r}\mathrm{g}{X}_{\mathrm{H}}-\mathrm{a}\mathrm{r}\mathrm{g}{Y}_{\mathrm{H}})}\right]\right|}{\mathrm{E}\left[\left|{X}_{\mathrm{H}}\right|\left|{Y}_{\mathrm{H}}\right|\right]}$$

### Base classifiers

For each set of connectivity matrices (frequency band × metric), a base classification model was trained using the Fisher’s Geodesic Minimal Distance to Mean (FgMDM) model^32^. As in the FUCONE method, this classifier can use a manifold distance to project sample SPD matrices onto the tangent space at the mean of the training samples via logarithmic mapping. Fisher’s Discriminant Analysis is then applied in tangent space for dimensionality reduction, after which the resulting data points are projected back onto the original manifold (inverse mapping). Finally, classification is performed using a Minimum Distance to Mean classifier, which assigns unseen samples to the nearest class mean.

As in the FUCONE method, we construct these classifiers to use the Riemannian manifold with the affine-invariant Riemannian distance metric

$${d}_{\mathrm{R}}\left({\mathbf{C}}_{1},\left.{\mathbf{C}}_{2}\right)\right.=\:{\left\lVert\mathrm{log}\left({\mathbf{C}}_{1}^{-\frac{1}{2}}{\mathbf{C}}_{2}{\mathbf{C}}_{1}^{-\frac{1}{2}}\right)\right\rVert}_{\mathrm{F}}$$

between FC matrices $\:{\mathbf{C}}_{1}$ and $\:{\mathbf{C}}_{2}$, with log denoting the matrix logarithm. To assess whether alternative distance metrics may perform better for certain types of FC metrics, we additionally explored two other distance metrics and their corresponding manifolds, the Riemannian log-Euclidean distance

$${d}_{\mathrm{L}}\left({\mathbf{C}}_{1},{\mathbf{C}}_{2}\right)={\left\lVert\mathrm{log}{\mathbf{C}}_{2}-\mathrm{log}{\mathbf{C}}_{1}\right\rVert}_{\mathrm{F}}$$

and the standard Euclidean distance

$${d}_{\mathrm{E}}\left({\mathbf{C}}_{1},{\mathbf{C}}_{2}\right)={\left\lVert{\mathbf{C}}_{2}-{\mathbf{C}}_{1}\right\rVert}_{\mathrm{F}}$$

### Stacked generalization ensemble classifiers

To construct the overall classification model, all base classifiers for pairs of frequency band and connectivity metrics were combined in a stacked ensemble. Stacked generalization^33^ is an ensemble machine learning technique in which a meta-classifier is trained on cross-validated predictions from base models, in our case the FgMDM classifiers for each pair of frequency band and connectivity metrics. Nested five-fold stratified cross validation was used to generate base predicted probabilities, thereby ensuring that segments corresponding to the same subject are assigned to the same fold. Only the class probability of the positive class was retained, as it is equal to one minus the class probability of the negative class.

**Fig. 1:**
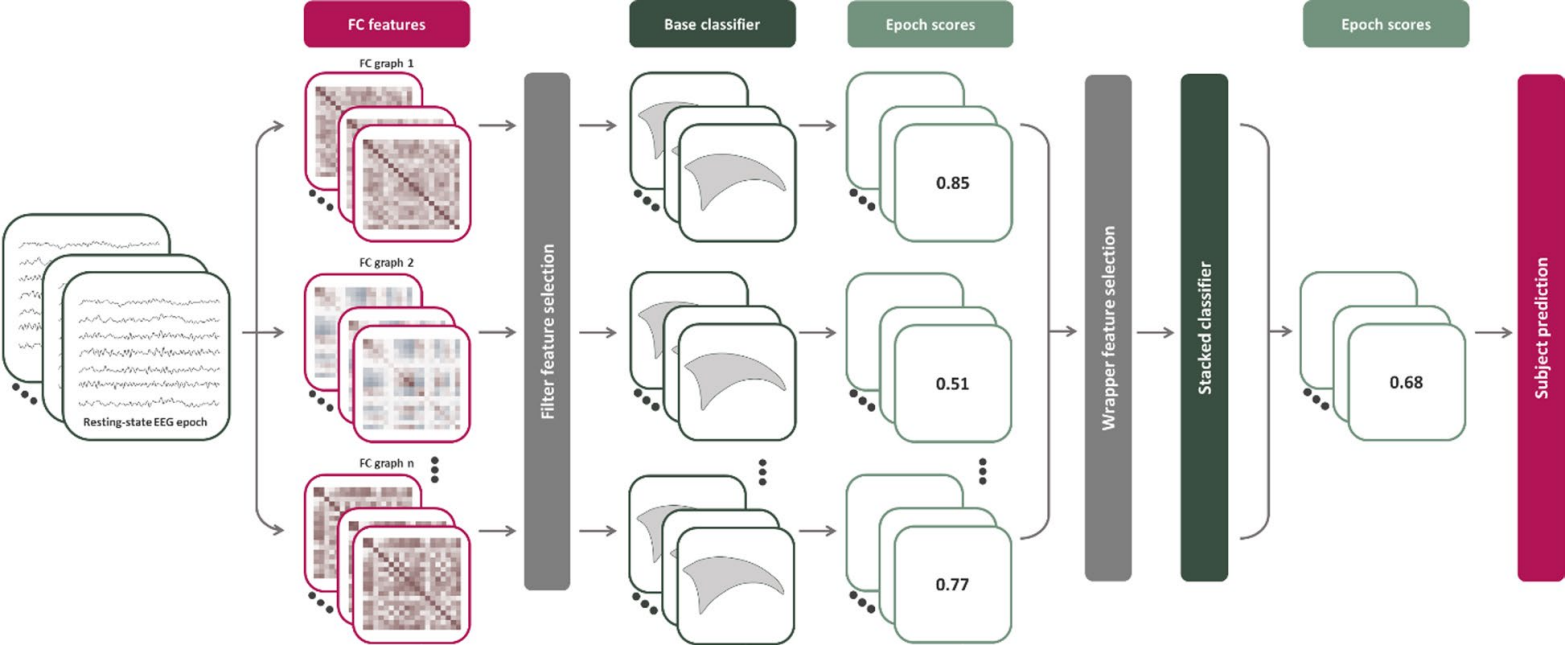
Outline of the proposed method. Resting-state EEG is segmented into epochs and filtered into multiple frequency bands. For each band, various functional connectivity metrics are extracted and scored by a base classifier. After feature selection, the stacked classifier generates the final epoch-level predictions based on the outputs of the base classifiers and aggregates these predictions to produce subject-level predictions. FC = Functional connectivity.

Since the meta-classifier predicts at the subject level, while base models produce epoch-level predictions, epoch probabilities predicted by the meta-classifier were averaged per subject. The meta-classifier, a logistic regression model with Elastic Net regularization, a weighted combination of *L*_1_ and *L*_2_ norm regularization solved for coefficients **w**:

$$\min_\mathbf{w}\sum\limits_{i}\left[\log\left(1 + e^{\mathbf{x}_i^{\mathbf{T}}\mathbf{w}}\right)- y_i\,\mathbf{x}_i^{\mathbf{T}}\mathbf{w}\right]+ \alpha \lVert \mathbf{w} \rVert_{1}+ \alpha(1-\lambda)\lVert \mathbf{w} \rVert_{2}^{2}$$

was then trained on these aggregated probabilities $\mathbf{X}$, with binary class vector y, regularization strength α, and *L*_1_-ratio λ . The regularization strengths were set to α = 1 and λ = 0.15 based on pilot experimentation. The complete outline of the proposed method is shown in Fig. 1.

### Feature selection

A first feature selection step before applying the stacked ensemble was implemented according to the filter paradigm. Each set of connectivity matrices extracted for a given frequency band and connectivity metric was scored based on class distinctiveness as defined by Lotte & Jeunet^34^, based on the corresponding distance metric. Supervised scoring was performed using the labels of the corresponding training fold. Matrix features were ordered using class distinctiveness and the top 50% were retained.

During the stacked ensemble training, the meta-classifier relies on the cross-validated predictions of the base classifiers to perform an additional wrapper feature selection step. Sequential feature selection iteratively includes the base classifier that performs best in the meta estimator based on internal, stratified 5-fold cross-validated ROC-AUC, in a greedy manner, until performance no longer improves.

### Model evaluation

The ensemble model was trained on connectivity matrix features obtained from epoched data, with each training sample corresponding to one epoch. The base classifiers and meta-classifier were trained with sample weights inversely proportional to the number of epochs for the corresponding subject, to account for the unequal number of epochs per subject. Performance of the ensemble model was evaluated using leave-one-subject-out cross-validation (LOSOCV), ensuring that all epochs from a given subject were assigned to the same fold to prevent data leakage. For each fold, all epochs from one subject were in the test set, while epochs from the remaining subjects constituted the training set. Within each LOSOCV training fold, the stacking ensemble additionally uses internal cross-validated prediction to generalize the meta-classifier. Specifically, each LOSOCV training fold is again split into five stratified folds, while ensuring that all epochs from a given subject remain within the same fold.

Performance metrics for subject-level classification were calculated from the cross-validated predicted class probabilities by averaging the epoch-level probabilities per subject to obtain a single class probability per subject. Where explicit predictions were needed instead of probabilities, a threshold of 0.5 was applied to these probabilities to determine the predicted class.

All evaluations were performed in a binary classification setting, classifying AD vs. HC, FTD vs. HC, or FTD vs. AD, with the former of each pair as the positive class. The following performance metrics were used: accuracy, sensitivity, specificity, F1 score, and area under the receiver operating characteristic curve (ROC-AUC).

Because accuracy is sensitive to class imbalance, we relied primarily on ROC-AUC and F1-score for objective evaluation of the problem. F1-score balances precision and recall across classes:

$$\mathrm{F}1=\frac{2\times\mathrm{P}\mathrm{r}\mathrm{e}\mathrm{c}\mathrm{i}\mathrm{s}\mathrm{i}\mathrm{o}\mathrm{n}\times\mathrm{R}\mathrm{e}\mathrm{c}\mathrm{a}\mathrm{l}\mathrm{l}}{\mathrm{P}\mathrm{r}\mathrm{e}\mathrm{c}\mathrm{i}\mathrm{s}\mathrm{i}\mathrm{o}\mathrm{n}+\mathrm{R}\mathrm{e}\mathrm{c}\mathrm{a}\mathrm{l}\mathrm{l}}$$

Lastly, ROC-AUC summarizes the model’s ability to discriminate between classes across different decision thresholds, providing a scalar performance measure.

### Ensemble diversity

To assess classifier diversity, normalized diversity was used as introduced by Heidemann et al.^35^, which expresses the proportion of samples on which two classifiers disagree, normalized by the expected disagreement given their joint accuracy. Higher values indicate greater diversity. Diversity of base classifiers is an important element of an ensemble classifier. If the base classifiers each produce different, uncorrelated errors, the bias of the predictions obtained from the meta-classifier will be reduced, improving robustness of the ensemble^36^. Furthermore, heavily correlated base classifier predictions cause multicollinearity in the meta-classifier training data, hampering stability and generalization^37^.

Normalized disagreement for a pair of classifiers $\:i$ and $\:j$ is calculated as

$${D}_{i,j}=\frac{{N}_ {\hat{\rm{y}}_{i}\ne \hat{\rm{y}}_{j}}}{N\left(1- {\mathrm{Accuracy}}_{i,j}\right)}$$

with $N$ the number of predictions, ${\hat{\rm{y}}_{i}\ne \hat{\rm{y}}_{j}}$ the number of predictions that are different between the classifiers, and $\mathrm{Accuracy}_{i,j}$ the accuracy of their ensemble.

## Results

### Contribution of functional connectivity metrics

**Fig. 2:**
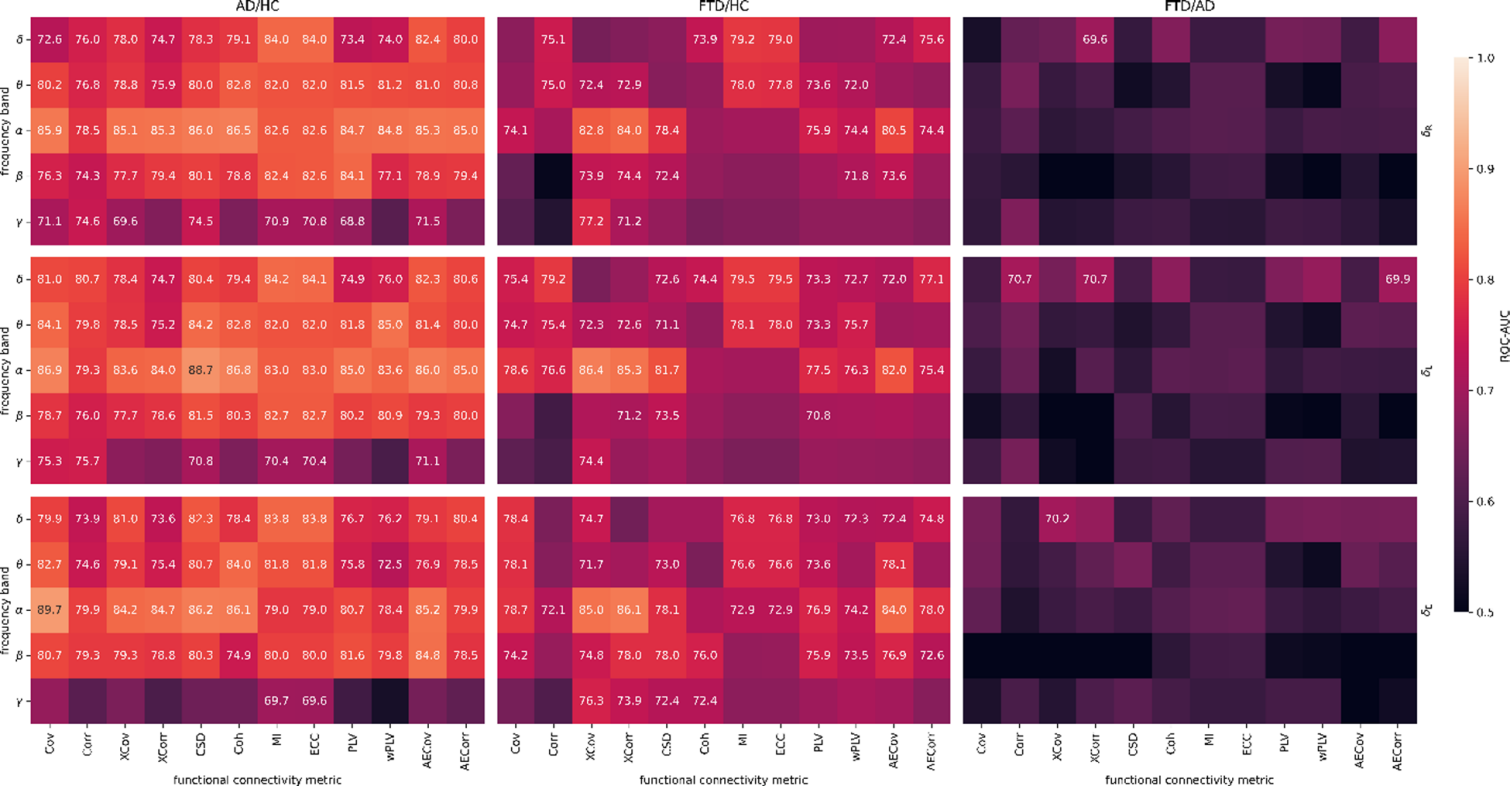
Classification performance of functional connectivity metrics. ROC-AUC values (%) obtained through LOSOCV are shown for base classifiers trained on each combination of frequency band (rows) and functional connectivity metrics (columns) for each classification problem and for each distance metric (affine-invariant Riemannian, log-Euclidean, and Euclidean shown in top, middle, and bottom rows, respectively). Only values reaching significance (DeLong test, α = 0.05) are annotated. AECorr = Amplitude envelope correlation, AECov = Amplitude envelope covariance, AD = Alzheimer’s disease, Coh = Coherence, Corr = Correlation, Cov = Covariance, CSD = Cross-spectral density, ECC = Entropy correlation coefficient, FTD = Frontotemporal dementia, HC = Healthy controls, MI = Mutual information, PLV = Phase locking value, ROC-AUC = area under the receiver operating characteristic curve, XCorr = Cross correlation, XCov = Cross covariance, wPLV = Weighted phase locking value.

To evaluate the contribution of FC metrics within different frequency bands, we trained a single base classification model for each combination of frequency band and connectivity metric and assessed performance using ROC-AUC (Fig. 2). Statistical significance was determined with DeLong tests. All distance metrics performed similarly, with a minor advantage for Log-Euclidean distance (Table 3), which was thus retained for further analyses. For the AD/HC problem, the best performance over all distance metrics was observed in the alpha band, with several connectivity metrics exceeding a ROC-AUC of 85%. The maximum ROC-AUC using log-Euclidean distance of 88.70% was observed for the cross-spectral density in the alpha band. For the FTD/HC problem, the best performance was also observed in the alpha band, specifically for cross-covariance using log-Euclidean distance (ROC-AUC = 86.36%). In distinguishing FTD from AD, the only significant FC metrics were found in the delta band, with cross-correlation reaching ROC-AUC = 70.65%. Best performing metrics for each problem are summarized in Table 1 (last row). As only three different metrics in the delta band reached significance across all base classifiers, this suggests that distinguishing between both dementias is the most challenging classification problem. The alpha band achieved the highest overall performance in distinguishing AD or FTD from HC, while delta band FC metrics performed best in distinguishing the two dementias from each other. Following FTD/AD results, only normalized FC metrics (Corr, XCorr, Coh, ECC, AECorr) and wPLV were retained to decrease the search space in further analyses.

**Table 3:** Average ROC-AUC values obtained per distance metric. Average of single-base classifier problems’ ROC-AUC obtained through LOSOCV were calculated as the mean of the values corresponding to all frequency bands and functional connectivity metrics presented in Fig. 2.

	AD/HC	FTD/HC	AD/FTD
Riemannian	78.24%	70.39%	57.85%
Log-Euclidean	78.84%	72.35%	58.26%
Euclidean	76.77%	73.20%	58.02%

AD = Alzheimer’s disease, FTD = Frontotemporal dementia, HC = Healthy controls.

### Pairwise classifier score and disagreement

**Fig. 3:**
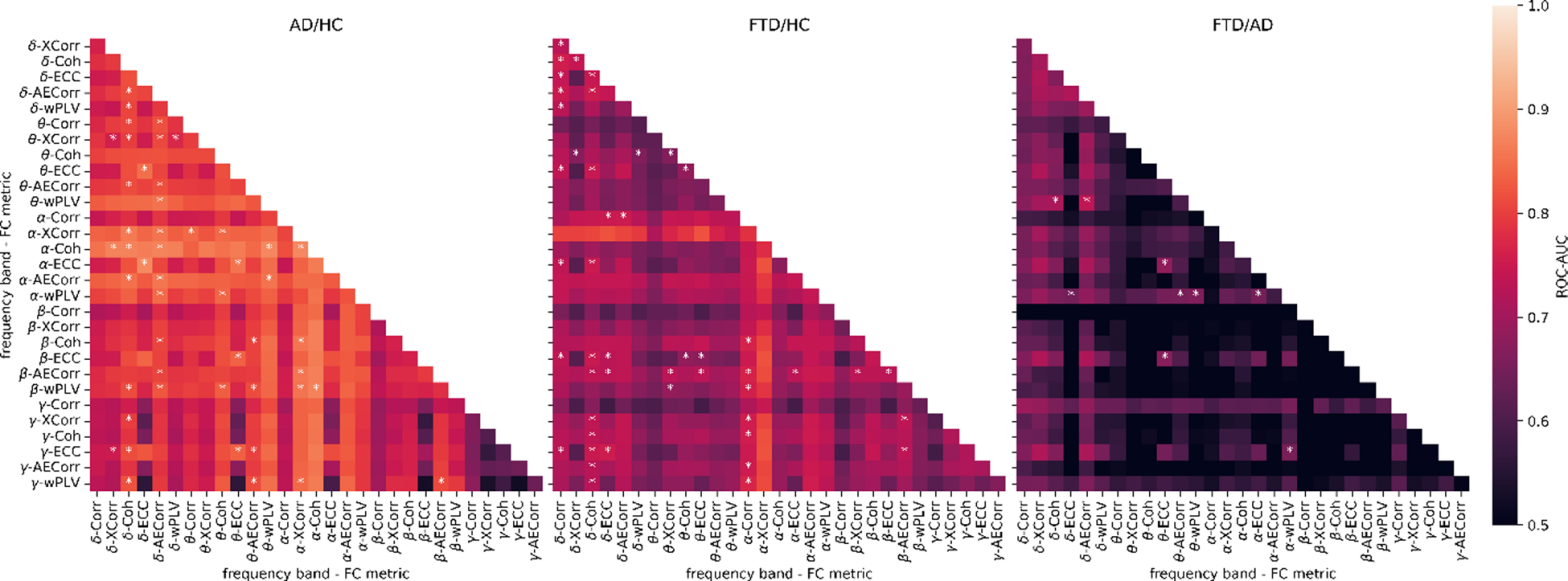
Pairwise stacked ensemble classification scores. Each cell reports the ROC-AUC score for a stacked ensemble of two base classifiers per classification problem (AD/HC, FTD/HC, FTD/AD). Stars indicate cases where the ensemble outperforms either of the single classifiers. AECorr = Amplitude envelope correlation, AD = Alzheimer’s disease, Coh = Coherence, Corr = Correlation, ECC = Entropy correlation coefficient, FTD = Frontotemporal dementia, HC = Healthy controls, ROC-AUC = area under the receiver operating characteristic curve, XCorr = Cross correlation, wPLV = Weighted phase locking value.

To evaluate whether stacking of base classifiers improves performance, we performed pairwise stacked ensemble analyses for each classification problem. For computational efficiency, we used stratified 5-fold cross-validation instead of LOSOCV, both for scoring the pairwise ensembles and the single classifiers used in the normalized disagreement calculation. Using stratified 5-fold cross-validation substantially reduced the computational demands while still accounting for subject groups, providing a realistic approximator of the performance for comparison and interpretation purposes. The results indicate that stacking improved performance for several metric pairs (Fig. 3). The highest pairwise ROC-AUC was obtained for the AD/HC classification problem (ROC-AUC = 87.84%) using a combination of delta and alpha entropy correlation coefficient, achieving performance comparable to the best single-base classifier. For the FTD/HC problem, the ensemble of beta entropy correlation coefficient and alpha cross-correlation features resulted in a ROC-AUC of 82.01%. For the FTD/AD problem, the best ensemble achieved a ROC-AUC of 73.55%, using the ensemble of delta cross-correlation and alpha entropy correlation coefficient, again reaching similar performance as the best single base classifier for this problem. Overall, while stacked ensembles outperformed either of the single classifiers in several cases, the overall improvement was generally modest, suggesting that stacked ensembles of two classifiers do not consistently lead to substantial improvement of classifier performance.

**Fig. 4:**
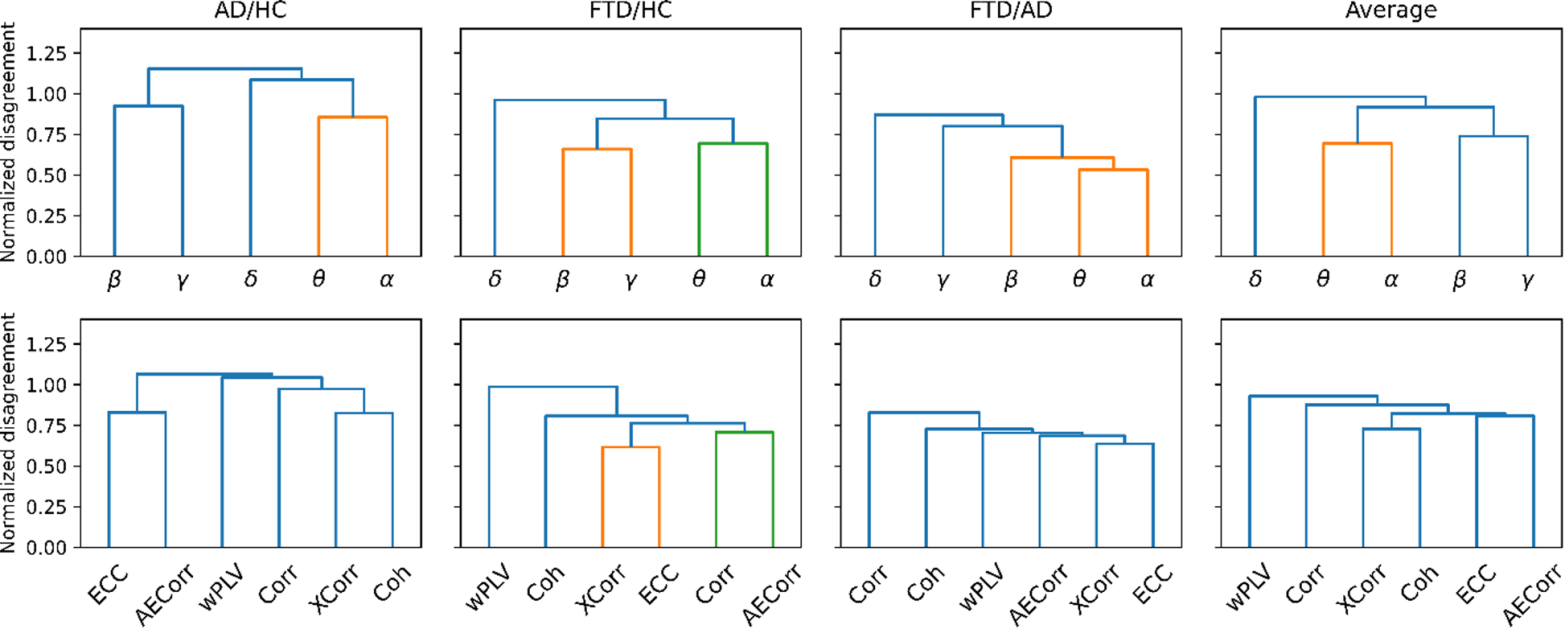
Normalized disagreement between base classifiers across classification problems. Hierarchical clustering dendrograms are shown for frequency bands (top) and different connectivity measures (bottom) separately for each classification problem and the average across problems. Hierarchic clusters with a threshold of 75% of the maximum disagreement are shown in distinct colors. Higher normalized disagreement indicates greater ensemble diversity. AECorr = Amplitude envelope correlation, AD = Alzheimer’s disease, Coh = Coherence, Corr = Correlation, ECC = Entropy correlation coefficient, FTD = Frontotemporal dementia, HC = Healthy controls, XCorr = Cross correlation, wPLV = Weighted phase locking value.

Next, we calculated the normalized disagreement between classifiers to quantify their diversity and identify which frequency bands and connectivity metrics provide redundant or complementary information. To identify redundant frequency bands or FC metrics, we performed a hierarchical cluster analysis of this normalized disagreement, with a relative clustering threshold at 75% of the maximum disagreement (Fig. 4), as implemented in SciPy (v1.6.2)^38^. Our results suggest that some features are closely related on average over all problems, reflecting properties of the underlying signal. In particular, theta and alpha frequency bands produced similar predictions, suggesting limited exploitable diversity between classifiers based on these bands (Fig. 4, top row). A structure with clear clusters, as observed in the average frequency disagreement, suggests that classifiers within these clusters are largely redundant for the respective problem, reflecting the structure of the underlying EEG signal or the definition of the FC metrics and resulting in low diversity. A flatter structure, as observed in the average FC metric panel, indicates that classifiers generally make different errors, although disagreement values tend to be lower for metrics with overall lower classification performance.

On average, no clusters were found for the FC metrics, indicating that they hold complementary information, irrespective of the classification problem. Interestingly, FC metrics were not necessarily clustered according to their domain (temporal or frequency). For example, cross-correlation was more closely related to coherence. Weighted PLV was the most complementary to other metrics.

The same hierarchical clustering analysis was used to select performant feature set for the binary classification problem, for subsequent stacking ensemble analysis. For the AD/HC problem, the gamma band was excluded due to its general low performance, and the theta band was excluded since it was shown to be complementary to the alpha band. For the FTD/HC problem, we excluded the gamma and theta frequency bands as well as entropy correlation and correlation. For FTD/AD, the beta and theta frequency bands were excluded from subsequent stacking ensemble analysis to focus on features that contributed more complementary information.

### Stacking ensemble

**Fig. 5:**
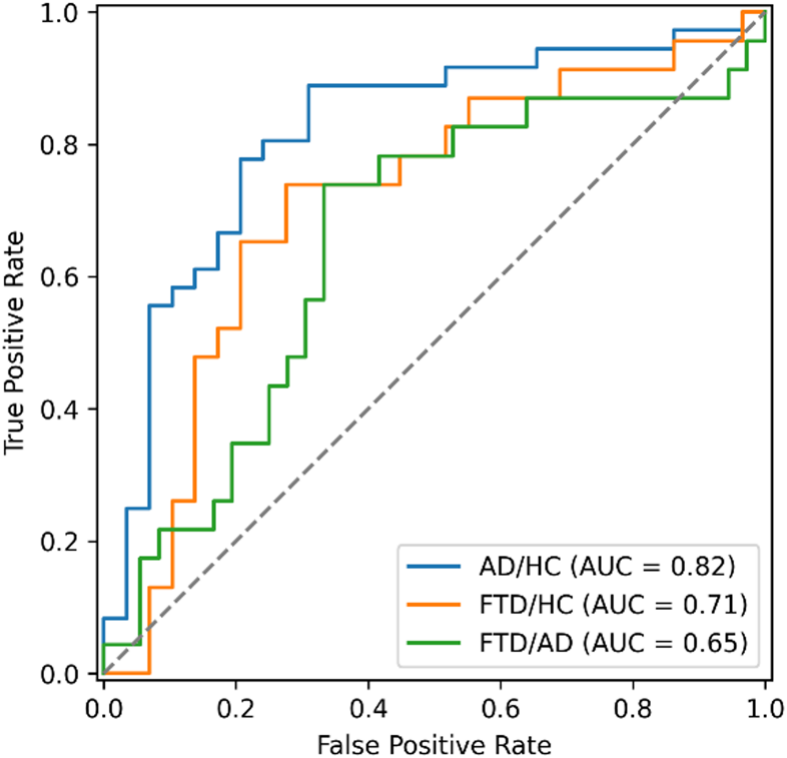
Receiver operating characteristic curves for the stacked ensemble classifiers. Overall performance is lowest for the FTD/AD classification problem. AD = Alzheimer’s disease, AUC = Area under the curve, FTD = Frontotemporal dementia, HC = Healthy controls.

While the pairwise stacked ensemble analysis highlighted several cases in which combining two classifiers resulted in a higher performance, these results were based on manually selected classifier pairs. To evaluate whether stacking yields more robust and generalizable improvements, we implemented a full ensemble combining all remaining base classifiers. As summarized in Table 1 (second-to-last row), the full ensemble achieved ROC-AUC values of 81.80% for AD/HC, 71.36% for FTD/HC, and 65.10% for AD/FTD using LOSOCV (Fig. 5). The model performed best in distinguishing AD and HC, whereas discriminating FTD and AD remained the most challenging, consistent with previous results. These outcomes indicate that, while the full stacked ensemble provides stable performance across all classification problems, it does not substantially exceed the best-performing individual or pairwise classifiers, suggesting that the remaining base models still share overlapping predictive information or the more complex approach leads to overfitting.

To further interpret the features selected by the ensemble model, we refit the model on the entire dataset, for each classification problem, without cross-validation. This allowed inspection of the features retained after the filter and wrapper feature selection steps and *L*_*1*_-regularization. Only a small number of features were ultimately retained by the ensemble (AD/HC: alpha coherence and weighted phase-locking value; FTD/HC: alpha and beta amplitude-envelope correlation; FTD/AD: alpha coherence). Notably, these features do not necessarily correspond to the best-performing single-base classifiers, which may partly explain the performance gap observed between single-classifier and ensemble results.

Lastly, we conducted two ablation studies to evaluate the importance of feature selection stages, one eliminating the early filter feature selection and another eliminating the wrapper feature selection. Omitting the filter feature selection led to increased ROC-AUC for AD/HC (+ 0.86% points) and FTD/HC (+ 6.74% points) but resulted in a pronounced decrease for FTD/AD (-10.75% points), which represents the most difficult and clinically relevant classification problem. This suggests that the overall procedure is sensitive to initial feature selection, particularly for FTD/AD discrimination, and that appropriate heuristics are required. Similarly, eliminating the sequential wrapper feature selection increased ROC-AUC for AD/HC (+ 1.27% points) and FTD/HC (+ 0.60% points), while decreasing FTD/AD classification performance (-6.65% points). Taken together, these findings indicate that stronger feature selection may be more relevant for an ensemble of weak classifiers, as is the case for the FTD/AD classification problem.

## Discussion

In this study, we aimed to classify between AD, FTD, and HC using several FC metrics extracted from resting-state EEG. We applied a stacked generalization ensemble approach and evaluated the importance of different frequency bands and connectivity metrics to classification performance.

With the stacked approach, our model achieved a ROC-AUC of 81.80% in distinguishing between AD and HC, and a ROC-AUC of 71.36% in distinguishing between FTD and HC. This was lower compared to a single base classifier performance, where several connectivity metrics achieved a ROC-AUC of 85% or higher for both classification problems. While these values reflect moderate discriminative ability, our results were obtained under a strict LOSOCV, providing a robust estimate of generalizability for a dataset of limited size. Even though the stacking approach did not outperform single base classifier performance, our approach allowed us to identify which FC metrics and frequency bands contributed most to classification. Alpha band connectivity achieved the highest overall performance in distinguishing both dementias from cognitively unimpaired individuals, with several FC metrics reaching a ROC-AUC of 85% or slightly above. Delta band achieved the highest performance in distinguishing between AD and FTD, with correlation and cross-correlation being the best performing metrics (ROC-AUC = 70.70%). This is in line with previous EEG studies consistently showing changes in peak frequency, power, and synchronization in alpha, delta, and theta bands related to AD^39,40^. Generalized EEG slowing is commonly reported in AD^6^, characterized by increased power in slow frequency bands (delta and theta) and a reduction in faster bands^7,10^. In particular, disruptions in alpha band synchronization have been suggested to reflect global network disconnection arising from synaptic loss and impaired long distance connections^41^. In contrast, qualitative EEG assessments in FTD typically show less prominent changes in the delta band and a decrease in higher frequencies^10^. In line with these observations, previous EEG FC studies have reported reduced FC in the delta band in AD compared to FTD^42^. Together, these findings may explain why alpha-band FC metrics achieved higher performance in separating AD and FTD from HC but were less specific for differentiating between AD and FTD. Another study using the same dataset similarly showed that delta, alpha, and gamma frequency bands were most discriminative frequency bands in coherence and cross-correlation^17^. Cross-correlation was among best performing single classifiers in the current study as well, especially in alpha and delta frequency bands. Together, these observations indicate that the large-scale FC metrics used in the current study capture meaningful neurophysiological changes, even if their discriminative power between dementia subtypes remains limited.

Distinguishing between AD and FTD showed to be the most difficult classification problem, with ROC-AUC of 65.10% using the stacked approach and ROC-AUC of 70.65% with the best single base classifier (cross-correlation in delta band). In contrast, separating each dementia from cognitively unimpaired individuals seemed to be more straightforward, as mentioned above. These results suggest that AD and FTD share some overlapping functional network disruptions, which might make them less separable using the same connectivity metrics that clearly distinguish them from controls. The FC metrics used in the current study likely capture shared neurophysiological changes in both AD and FTD, rather than disease specific changes uniquely characterizing each dementia. Previous studies have suggested that EEG changes in AD and FTD may follow distinct spatial distributions, with FTD showing changes between the frontal and temporal lobes^9^. While our approach estimated FC between EEG channels across several regions, the use of a standard 19-channel montage may not have provided sufficient spatial resolution to capture these region-specific differences. Future studies using higher-density EEG or source-localized connectivity analyses may better capture these disease-specific network alterations.

While previous studies have reported similar^16,27^ or even higher^14,17,23,24,26^ classification performance when distinguishing FTD from AD, direct comparison is difficult due to differences in preprocessing, the level of analysis (epoch vs. subject level), feature selection (inclusion of demographic information), and validation strategy (fixed test splits, k-fold, LOSOCV, or stratification). Studies with the highest-performing models typically used test-split or epoch-level cross-validation to evaluate their method, which may overestimate performance depending on the size and composition of the test split. This issue was already raised by Miltiadous et al.^18^  where authors showed a significant drop in model performance when they used LOSOCV instead of ten-fold cross validation on the epoched data. Another methodological limitation is that it is not always clear whether previous studies classified individual epochs and reported those results directly, or whether predictions were aggregated per subject. Considering that not all epochs from a given subject may be consistently classified into the same category, this distinction can have a large impact on reported performance metrics. In the present study, all classifiers were evaluated on their ability to predict the class of individual subjects by averaging the predicted probabilities across all epochs belonging to the same subject, thereby ensuring subject-level evaluation and eliminating potential data leakage. In contrast to the above-mentioned studies, we used LOSOCV to interpret FC features, providing a more robust assessment of performance given a dataset with few samples, since reported metrics are not dependent on an arbitrary chosen test set or small cross-validation test folds. We therefore call for consistent performance reporting to enable reproducibility, meta-analyses, and fair comparison across studies using this dataset. In our view, the most appropriate methodology for this specific dataset is to produce a single prediction or probability per subject using LOSOCV. When analyses are performed at the epoch level, all epochs from a given subject should be confined to a single split, both in performance estimation and in potential inner cross-validation used for hyperparameter tuning.

Our stacked ensemble approach did not show improvement over the best single base classifiers. This could be explained by the limited diversity observed between base classifiers, sometimes combined with their relatively low baseline performance. Normalized disagreement analysis showed that several base classifiers produced similar predictions. Based on this, clusters of similar metrics were identified (Fig. 4), such as theta and alpha frequency bands for all classification problems. This indicates that these features provided overlapping rather than complementary information, likely limiting the potential benefit of a stacking ensemble, which usually benefits from diverse base models^22,35^. However, the FC metrics generally exhibited large disagreement.

Although Riemannian geometry is proposed to more accurately account for intrinsic manifold geometry of SPD matrices, we observed no significant benefits of Riemannian over Euclidean distance in our study. Classifiers based on the Euclidean distance metric generally achieved comparable performance to the affine-invariant Riemannian and the log-Euclidean distance (Table 3), suggesting limited or no practical benefits in this classification context. This can be explained by two arguments. First, the robustness of the affine-invariant Riemannian distance, stemming from its affine invariant property, and the scale invariance of the log-Euclidean metric, are suitable for covariance matrices, since covariance directly reflects linear relationships in the EEG signal^43^. However, many non-linear connectivity measures do not necessarily share these properties and therefore may not benefit from such geometric formulations other than the geodesic convexity of the SPD manifold.

Second, manifold approaches are typically more effective in within-subject classification problems, where multiple classes or trials are available per subject, or in within-subject transfer learning across subjects. The tangent space projection used in the FgMDM classifier and other Riemannian classifiers is based on locality^43^. In between-subject problems, clusters of epochs belonging to different subjects are not necessarily local to each other and it might be difficult to fit a single tangent space common to all epochs^43^. When all epochs from a given subject belong to the same class, as is the case in the diagnostic problem at hand, the tangent space might not be a good fit and the contribution of Riemannian distance metrics might be limited.

Even though our approach reached moderate classification performance, our findings support the potential of resting-state EEG as a scalable tool for neurodegenerative disorders. Furthermore, we show that single, well-chosen features combined with a direct analysis pipeline achieve more generalizable performance than aggregating an arbitrary range of multiple metrics. This highlights the importance of careful feature engineering when working with limited datasets. Future work should focus on the identification of robust and interpretable criteria, for example by combining well-performing EEG connectivity metrics with other biomarkers, such as structural MRI measures, CSF or plasma biomarkers, domain-specific cognitive measures (e.g., executive or memory performance), or longitudinal EEG recordings to improve diagnostic specificity, rather than increasing model complexity. Additionally, variability across epochs and temporal stability of the signal could be assessed, to evaluate whether temporal dynamics provide additional information beyond subject-level average across epochs. Lastly, test-retest stability of different FC metrics could be assessed to identify metrics that remain reliable across subjects in small clinical datasets. Together, these results emphasize that simplicity and interpretability can offer stronger generalizability than complex ensembles in EEG-based dementia classification.

## Data Availability

The dataset analyzed during the current study is available in the OpenNeuro repository^13^, https://doi.org/10.18112/openneuro.ds004504.v1.0.5.
