# Correction: EEG-based classification of alzheimer’s disease and frontotemporal dementia using functional connectivity

**DOI:** 10.1038/s41598-026-49348-8

**Published:** 2026-05-13

**Authors:** Tjaša Mlinarič, Arne Van Den Kerchove, Zoe I. Barinaga, Marc M. Van Hulle

**Affiliations:** 1 Laboratory for Neuro- and Psychophysiology, Department of Neurosciences, KU Leuven, Leuven, Belgium; 2 Leuven Brain Institute, KU Leuven, Leuven, Belgium; 3 University of Lille, CNRS, Centrale Lille, Lille, France

Correction to: *Scientific Reports* 10.1038/s41598-026-35316-9, published online 09 January 2026

In the original PDF version of this Article, four equations did not display correctly.

In the Methods section, under the subheading ‘Information domain metrics’,

“When modeling $X$ and $Y$ as stochastic variables, their entropy $\mathrm{H}\left(X\right)$ and conditional entropy $\mathrm{H}\left(X?Y\right)$ can be determined. Their mutual information is then defined as

$$\mathrm{MI}\left(X,Y\right)=\mathrm{H}\left(X\right)-\mathrm{H}\left(X?Y\right)."$$

now reads:

“When modeling $X$ and $Y$ as stochastic variables, their entropy $\mathrm{H}\left(X\right)$ and conditional entropy $\mathrm{H}\left(X|Y\right)$ can be determined. Their mutual information is then defined as

$$\mathrm{MI}\left(X,Y\right)=\mathrm{H}\left(X\right)-\mathrm{H}\left(X|Y\right)."$$

Under the subheading ‘Ensemble diversity’,

“Normalized disagreement for a pair of classifiers $\:i$ and $\:j$ is calculated as

$$\:{D}_{i,j}=\frac{{N}_{{?}_{i}\ne?_{j}}}{\:N\left(1-\mathrm{Accuracy}_{i,j}\right)}$$

with $N$ the number of predictions, ${?}_{i}\ne{?}_{j}$ the number of predictions that are different between the classifiers, and $\mathrm{Accuracy}_{i,j}$ the accuracy of their ensemble.”

now reads:

“Normalized disagreement for a pair of classifiers $\:i$ and $\:j$ is calculated as

$$\:{D}_{i,j}=\frac{{N}_{\hat{y}_{i}\ne \hat{y}_{j}}}{\:N\left(1-\mathrm{Accuracy}_{i,j}\right)}$$

with $N$ the number of predictions, $\hat{y}_{i}\ne \hat{y}_{j}$ the number of predictions that are different between the classifiers, and $\mathrm{Accuracy}_{i,j}$ the accuracy of their ensemble.”

The PDF version of the original Article has been corrected.

